# Intestinal microbiota and anastomotic leakage of stapled colorectal anastomoses: a pilot study

**DOI:** 10.1007/s00464-015-4508-z

**Published:** 2015-09-18

**Authors:** Jasper B. van Praagh, Marcus C. de Goffau, Ilsalien S. Bakker, Hermie J. M. Harmsen, Peter Olinga, Klaas Havenga

**Affiliations:** Department of Surgery, University Medical Center Groningen, University of Groningen, Hanzeplein 1, PO Box 30.001, 9700 RB Groningen, The Netherlands; Department of Medical Microbiology, University Medical Center Groningen, University of Groningen, Groningen, The Netherlands; Pharmaceutical Technology and Biopharmacy, Department of Pharmacy, University of Groningen, Groningen, The Netherlands

**Keywords:** Anastomotic leakage, Intestinal microbiome, Colorectal surgery, Stapled anastomosis, DNA sequencing, Complications

## Abstract

**Background:**

Anastomotic leakage (AL) after colorectal surgery is a severe complication, resulting in morbidity, reinterventions, prolonged hospital stay and, in some cases, death. Some technical and patient-related aetiological factors of AL are well established. In many cases, however, none of these factors seem to explain the occurrence of AL. Recent studies suggest that the intestinal microbiome plays a role in wound healing, diabetes and Crohn’s disease. The aim of this study was to compare the intestinal microbiota of patients who developed AL with matched patients with healed colorectal anastomoses.

**Methods:**

We investigated the microbiome in the doughnuts collected from 16 patients participating in the C-seal trial. We selected eight patients who developed AL requiring reintervention and eight matched controls without AL. We analysed the bacterial 16S rDNA of both groups with MiSeq sequencing.

**Results:**

The abundance of *Lachnospiraceae* is statistically higher (*P* = 0.001) in patient group who did develop AL, while microbial diversity levels were higher in the group who did not develop AL (*P* = 0.037). Body mass index (BMI) was also positively associated with the abundance of the *Lachnospiraceae* family (*P* = 0.022).

**Conclusion:**

A correlation between the bacterial family *Lachnospiraceae*, low microbial diversity and anastomotic leakage, possibly in association with the BMI, was found. The relative abundance of the *Lachnospiraceae* family is possibly explained by the higher abundance of mucin-degrading *Ruminococci* within that family in AL cases (*P* = 0.011) as is similarly the case in IBD.

**Electronic supplementary material:**

The online version of this article (doi:10.1007/s00464-015-4508-z) contains supplementary material, which is available to authorized users.

Anastomotic leakage (AL) after colorectal surgery is a severe complication, resulting in morbidity, reinterventions, prolonged hospital stay and, in some cases, death. AL can be defined as a defect of the integrity of the intestinal wall at the anastomotic site leading to a communication of the intra- and extraluminal compartments. It may present as a subclinical abscess that drains spontaneously and needs no further treatment to a completely dehiscent anastomosis leading to a faecal peritonitis and sepsis. In many cases, a temporary or definitive ostomy is made. A nationwide Dutch audit revealed an AL rate (requiring reintervention) of 12 % in primary colorectal anastomoses and 9 % in anastomoses with a deviating ostomy [1]. In the literature, AL rates after colorectal surgery are reported in the range of 4–20 % [2].

Some aetiological factors of AL are well established. The anastomosis may be poorly constructed, with tension between the afferent and efferent loop, insufficient circulation or incomplete doughnuts. Healing of the anastomosis may be compromised, as could be the case in patients with diabetes, atherosclerosis or corticosteroid use. However, in many cases, none of these factors seem to explain the occurrence of AL. Therefore, it remains difficult to predict the occurrence of post-operative AL for the individual patient.

Recent studies showed that the composition of the bacterial growth in the intestine influences various processes in the body. For example, bacteria in the intestine are known to influence wound healing [3], and the intestinal microbiome has recently been linked to the origin of diabetes [4, 5]. The development of chemotherapy-induced mucositis is associated with an altered intestinal microbiome [6]. Even the recurrence of Crohn’s disease after resection is suggested to be under influence of microbes [7]. There is also a strong suggestion that the composition of the intestinal microbes affects the healing of the anastomosis and might hence be influenced by antibiotics [8]. In addition, selective decontamination of the digestive tract reduces infections and appears to have a beneficial effect on AL in colorectal surgery [9]. However, there are no publications relating the intestinal bacterial growth with surgical outcome of colorectal resections.

We hypothesized that the composition of the intestinal microbiome could play a significant role in anastomotic healing and the occurrence of leakage. The aim of this study was to compare the intestinal microbiota of patients who developed AL with matched patients with healed colorectal anastomoses, without clinical signs of AL.

## Materials and methods

### Patients

For this study, eight patients who developed AL requiring reintervention were selected and matched with eight patients without AL. Matching was done on gender, age and pre-operative chemotherapy and radiotherapy. All patients were included in the C-seal trial [2]. This multicentre trial was designed to evaluate the efficacy of the C-seal; the primary endpoint was AL requiring reintervention. This trial was open for inclusion from December 2011 until January 2014. Inclusion criteria were elective colorectal surgery with a circular stapled colorectal anastomosis, age ≥18 years, ASA score <4, mechanical pre-operative bowel preparation and no clinical signs of peritonitis. Exclusion criteria were major surgical or interventional procedures in the 30 days prior to this surgery or other interventional procedures planned within 30 days of entry in this study, and psychological, familial, sociological or geographical conditions which could potentially hamper compliance with the study protocol or the follow-up schedule.

The study was approved by the Medical Ethics Committee of the University Medical Center Groningen—University of Groningen and all participating centres and registered in the Dutch Trial Registry under the number NTR3080. All the patients provided written informed consent. All data were collected anonymously, encoded and saved in a database.

### Sample collection

For all patients who consented to be enrolled in this study, we retrieved and stored the ‘doughnut’. This ‘doughnut’ is the small ring of colon and rectum that is cut by the circular stapler to make the anastomosis. Bacterial DNA was isolated from the doughnut and was subsequently analysed using MiSeq sequencing to see whether the microbial composition could be linked with clinical outcome.

### DNA extraction

Total DNA was extracted from 0.25 g of a ‘doughnut’ using the repeated bead-beating method described in detail by Yu and Morrison [10], with a number of modifications. In brief, four 3-mm instead of 0.5-mm glass beads were added during the homogenization step. Bead beating was performed using a Precellys 24 (Bertin Technologies, Montigny-le-Bretonneux, France) at 5.5 beats per millisecond in three rounds of 1 min each with 30-s pauses at room temperature in between. The incubation temperature after the bead beating was raised from 70 to 95 °C. Importantly, protein precipitation with 260 μl of ammonium acetate was carried out twice instead of only once. Additional purification steps using columns were not needed after DNA precipitation.

### MiSeq preparation sequencing pipeline

The V3–V4 region of the 16S rRNA gene was amplified from the bacterial DNA by polymerase chain reaction (PCR) using modified 341F and 806R primers (Supplementary Table 1) with a six-nucleotide barcode on the 806R primer as described elsewhere [11, 12]. Reaction conditions consisted of an initial 94 °C for 3 min followed by 32 cycles of 94 °C for 45 s, 50 °C for 60 s and 72 °C for 90 s, and a final extension of 72 °C for 10 min. An agarose gel confirmed the presence of product (band at ~465 base pairs) in successfully amplified samples. The remainder of the PCR product (~45 μl) of each sample was mixed thoroughly with 25 μl Agencourt AMPure XP magnetic beads and was incubated at room temperature for 5 min. Beads were subsequently separated from the solution by placing the tubes in a magnetic bead separator for 2 min. After discarding the cleared solution, the beads were washed twice by resuspending the beads in 200 μl freshly prepared 80 % ethanol, incubating the tubes for 30 s in the magnetic bead separator and subsequently discarding the cleared solution. The pellet was subsequently dried for 15 min and resuspended in 52.5 μl 10 mM Tris HCl pH 8.5 buffer. Fifty microlitres of the cleared-up solution is subsequently transferred to a new tube. The DNA concentration of each sample was determined using a Qubit^®^ 2.0 fluorometer (www.invitrogen.com/qubit), and the remainder of the sample was stored at −20 °C until library normalization. Library normalization was done the day before running samples on the MiSeq by making 2 nM dilutions of each sample. Samples were pooled together by combining 5 μl of each diluted sample. Ten microlitres of the sample pool and 10 μl 0.2 M NaOH were subsequently combined and incubated for 5 min to denature the sample DNA. To this, 980 μl of the HT1 buffer from the MiSeq 2 × 300 kit was subsequently added. A denatured diluted PhiX solution was made by combining 2 μl of a 10 nM PhiX library with 3 μl 10 mM Tris HCl pH 8.5 buffer with 0.1 % Tween 20. This 5 μl mixture was mixed with 5 μl 0.2 M NaOH and incubated for 5 min at room temperature. This 10 μl mixture was subsequently mixed with 990 μl HT1 buffer. One hundred and fifty microlitres of the diluted sample pool was combined with 50 μl of the diluted PhiX solution and was further diluted by adding 800 μl HT1 buffer. Six hundred microlitres of the prepared library was loaded into the sample loading reservoir of the MiSeq 2 × 300 cartridge.

### MiSeq sequencing pipeline and statistical analysis

Software that was used to analyse the data received from Illumina paired-end sequencing, included PANDAseq [13], QIIME and ARB [14]. Reads with a quality score lower than 0.9 were discarded by PANDAseq. Statistical analyses were performed on the family, genus and species level. QIIME identified sequences down to the family and genus level and was used to perform weighted alpha-diversity analyses, while ARB was used to identify sequences down to the species level. Principal component analysis (PCA) was performed to describe the variation in all of the bacterial groups into a very limited amount of new relevant dimensions of variability in order to address the issue of multiple testing. In this study only, principal component 1, which describes over 67 % of the variation in the data, was correlated with the occurrence of AL. The hierarchical clustering analysis was performed with the Hierarchical Clustering Explorer rmed (http://www.cs.umd.edu/hcil/multi-cluster/). The Simpson index was used as a measure of microbial diversity. Nonparametric tests were used, as microbial abundances are rarely normally distributed. Mann–Whitney *U* or Spearman’s *ρ* tests were used as indicated. The use ± indicates that a standard deviation is given. All tests were two-tailed, and a *P* < 0.05 was considered to indicate statistical significance. All statistical analyses were performed using *IBM*^*®*^*SPSS*^*®*^*Statistics* 20.0.

## Results

The doughnuts of eight patients with AL and eight patients without AL were analysed. Patient characteristics are listed in Table 1. Body mass index (BMI) was slightly higher in the group of patients with AL, but was not a significant or independent factor for AL in this study group (*P* = 0.074, Mann–Whitney *U* test).

**Table 1:** Patient characteristics

	Anastomotic leakage (*n* = 8)	Control (*n* = 8)
Gender		
Male	7	7
Female	1	1
Age: min–max (mean) in years	57–75 (66.5)	57–75 (66.5)
Surgical indication		
Colorectal cancer	8	7
Diverticulitis	–	1
Preoperative treatment		
Chemotherapy	1	2
Radiotherapy	2	1
Body mass index (BMI) (kg/m^2^)	30.1	25.4

The microbial composition was successfully determined of 15 of the doughnuts. In one doughnut of a patient in the control group, the microbial identification was not successful, probably due to insufficient extraction of bacterial DNA.

### Bacterial composition in relation to AL

The strongest and most straightforward correlation that could be found between the bacterial composition and AL was that the abundance of the *Lachnospiraceae* family (27.3 ± 15.9 %) was significantly higher in patients who developed AL as compared to patients who did not develop AL (*P* = 0.001, Mann–Whitney *U* test). The most predominant genera of the *Lachnospiraceae* family (Ruminococcus (6.1 ± 11.9 %), Blautia (5.2 ± 4.5 %), Roseburia (4.4 ± 4.3 %) and Coprococcus (4.4 ± 4.4 %)) all contributed to this particular association. A receiver operator characteristic (ROC) curve showed that the sensitivity and specificity for the clinical outcome of this finding are high (as can be seen in the Supplementary Fig. 1). BMI was also positively associated with the abundance of the *Lachnospiraceae* family (*P* = 0.022, Spearman’s *ρ* test).

**Fig. 1:**
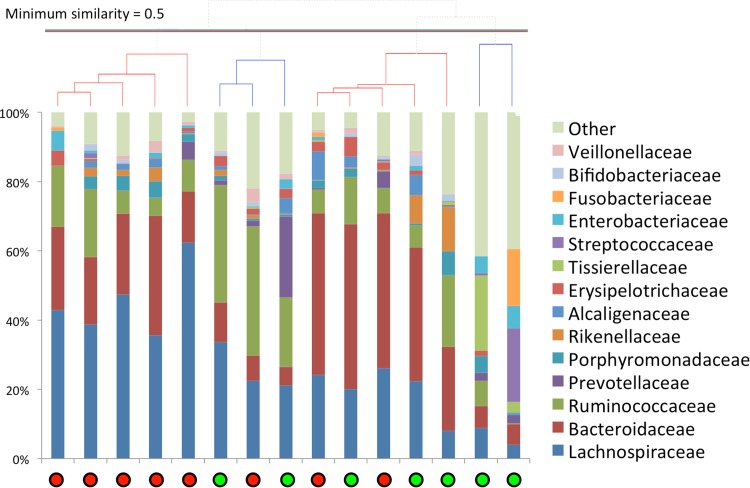
Hierarchical clustering analysis (*top*) in combination with the relative abundances of the different microbial families in samples from patients in whom AL occurred (*red circles*) and of those with no AL (*green circles*) developed (Color figure online)

Complete linkage clustering analysis of the sample on the family level furthermore identified one particular cluster of five samples from patients who developed AL who could be distinguished from the other samples mainly by having the highest *Lachnospiraceae* abundances of all samples. Samples not within this particular cluster (left cluster, Fig. 1) had lower *Lachnospiraceae* abundances (*P* = 0.002, Mann–Whitney *U* test).

**Fig. 2:**
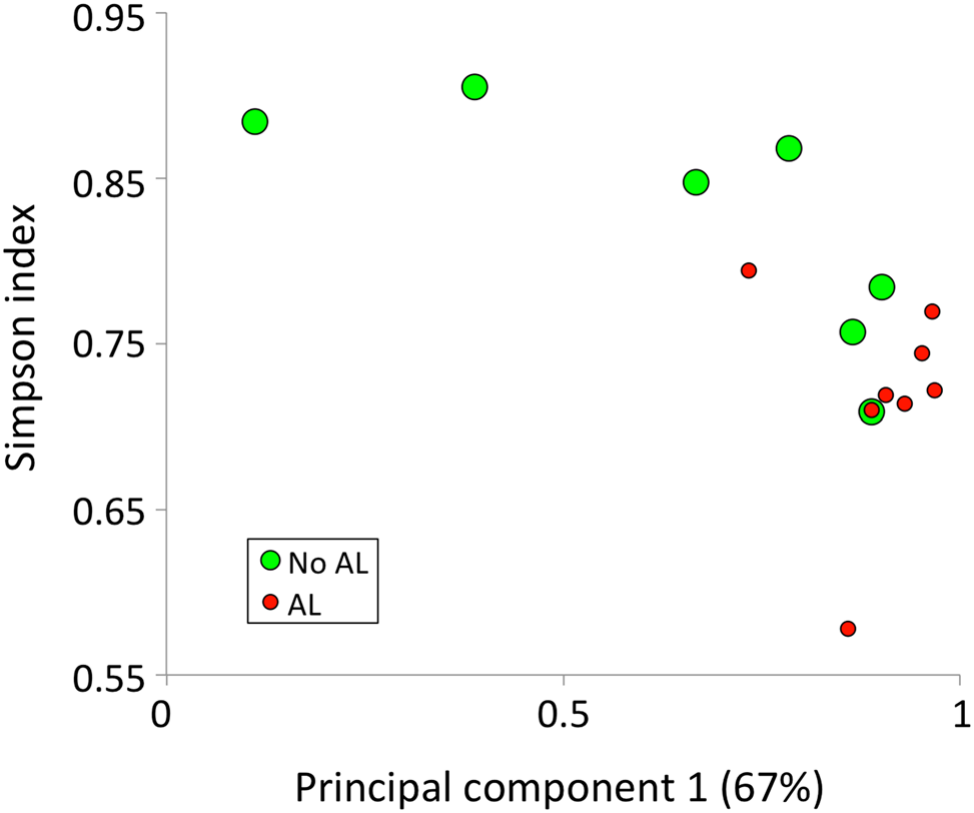
Principal component analysis (PC1, *x*-axis) in combination with a diversity analysis (*y*-axis) with respect to the occurrence (*red circles*) or absence (*green circles*) of AL in patients. AL is in general associated with a high score on PC1 and/or a low microbial diversity (Color figure online)

Principal component and alpha-diversity analyses on the family level, however, put the *Lachnospiraceae* association in a slightly different perspective. A principal component analysis shows that principal component 1 (PC1), which accounts for 67 % of the variation within the data, is positively correlated with patients developing AL (*P* = 0.021, Mann–Whitney *U* test), while the Simpson index, a measure of within (*α*) sample diversity, is negatively correlated with developing AL (*P* = 0.037, Mann–Whitney *U* test). Together, these two variables separate most AL and non-AL patients from one another (Fig. 2). Both the Simpson index and PC1 are strongly associated with the abundance of the two most abundant families, *Lachnospiraceae* and *Bacteroidaceae* (23.8 ± 15.5 %). The *Lachnospiraceae* abundance is positively correlated with PC1 (*P* = 0.003, Spearman’s ρ test) and negatively with the Simpson index (*P* = 0.007, Spearman’s ρ test), and the same is true for *Bacteroidaceae*. (*P* = 0.017 and *P* = 0.015, respectively). As earlier, a ROC curve showed here as well that the sensitivity and specificity of these tests are high, despite the group of only 16 patients (see Supplementary Fig. 2).

When dissecting the *Lachnospiraceae* finding (37 vs. 17 % average abundance, *P* = 0.001) down to the species level, it is found that much of the *Lachnospiraceae* pattern can be attributed to the variation in the amount of *Ruminococcus obeum*, a mucin-degrading bacterium (6.5 vs. 1.7 % average abundance, *P* = 0.021). Mucin-degrading *Ruminococci* from the *Lachnospiraceae* family as a whole, which include *R. gnavus* and *R. torques*, represent the most compelling suggestion for a clinically relevant finding, as their abundance is higher in AL cases (15.5 vs. 3.8 % average abundance, *P* = 0.011). The BMI, however, was only associated (yet not significantly) with *R. obeum* (*P* = 0.068), but not with the other *Ruminococci* or with any of the other individual bacterial species.

## Discussion

This pilot study on the possible role of the intestinal microbiota in the development of AL after colorectal resection with stapled anastomosis revealed interesting patterns. The correlation that was found between AL and the abundance of *Lachnospiraceae* (Fig. 1) was of particular interest as the association between the *Lachnospiraceae* family and AL was unexpected: most of the bacteria from this family are not particularly known to have a negative influence on the bowel. In fact, many butyrate-producing genera are found within the *Lachnospiraceae* family.

Butyrate is thought to be beneficial as it is the main energy source for colonic epithelial cells [15]. Furthermore, butyrate has been shown to regulate the assembly of tight junctions and to correlate with reduced gut permeability [16]. It also decreases intestinal inflammation by reducing oxidative stress in the colonic mucosa [17]. The Roseburia genus in particular is a well-known butyrate producer, which, similar to Faecalibacteria, is associated with protection against inflammatory bowel diseases [18]. However, a large fraction of the *Lachnospiraceae* reads was identified on the species level to be of mucin-degrading *Lachnospiraceae* (*R. obeum*, *R. gnavus* and *R. torques*) [19, 20]. The abundance of these mucin-degrading bacteria is commonly observed to be elevated in various inflammatory bowel diseases, such as Crohn’s disease, ulcerative colitis or irritable bowel syndrome [21–24].

On closer inspection, the association between *Lachnospiraceae* and AL could also be the result of the association between obesity and a lower microbial diversity. Obesity is known to be associated with a lower microbial diversity and with a low-grade systemic inflammation [25–27]. In addition, patients with an inflammatory bowel disease are known to have a low microbial diversity in the gut [27]. Besides that, a high BMI is associated with the development of AL [28–30]. Though the number of obese individuals in this study was limited, an association was found between BMI and *Lachnospiraceae* levels. *Lachnospiraceae* levels were strongly negatively correlated with microbial diversity levels that are in turn associated with AL (Fig. 2). So, the overabundance of *Lachnospiraceae* (or *Bacteroidaceae* is some cases) might not necessarily be directly linked with the development of AL, but with the absence of other (beneficial) microbial groups.

Alternatively, *Lachnospiraceae* could also be directly linked with AL as an increase in Firmicutes, of which *Lachnospiraceae* are an important member, is commonly found in obese people [25]. Butyric acid is also associated with obesity [31]. While butyrate is commonly associated with many beneficial effects stated earlier, an excess of butyrate might present the body with an excess of energy.

It could also be hypothesized that a poorly diversified microbiome is less stable than a well-diversified microbiome. The administration of prophylactic intravenous antibiotics, for instance, as is routinely done in colorectal surgery may cause larger shifts in the bacterial population in a poorly diversified microbiome, offering the opportunity for pathogenic bacteria to repopulate the lumen. The findings of Ohigashi et al. [32] contribute to this theory, as they found that after colorectal surgery the amount of possible pathogenic bacteria, as *Enterobacteriaceae, Enterococcus, Staphylococcus* and *Pseudomonas,* was significantly increased. We do not know, however, whether this also happened during the development of AL in our patients.

The main limitation of this study is the small number of included patients. Beside that, we only investigated AL and obesity in relation to the intestinal microbiota. In a follow-up study, we plan to include a much larger group of C-seal trial patients and perform a more detailed analysis of patient and treatment factors in relation to the intestinal microbiome.

## Electronic supplementary material

Below is the link to the electronic supplementary material.

Supplementary material 1 (DOCX 129 kb)

Supplementary material 2 (TIFF 72 kb)

Supplementary material 3 (TIFF 108 kb)
