# Supplementary material for: Intestinal microbiota and anastomotic leakage of stapled colorectal anastomoses: a pilot study
Source: Surg Endosc. 2015 Sep 18;30:2259–65. doi: 10.1007/s00464-015-4508-z (PMC4887536; doi:10.1007/s00464-015-4508-z)
Supplement: Supplementary file 1 — Supplementary material 1 (DOCX 129 kb) [file 464_2015_4508_MOESM1_ESM.docx]

| V3_F_modified | aatgatacggcgaccaccgagatctacactctttccctacacgacgctcttccgatctNNNNCCTACGGGAGGCAGCAG |
| --- | --- |
|  |  |
| V4_1R | caagcagaagacggcatacgagat**CGTGAT**gtgactggagttcagacgtgtgctcttccgatctGGACTACHVGGGTWTCTAAT |
| V4_2R | caagcagaagacggcatacgagat**ACATCG**gtgactggagttcagacgtgtgctcttccgatctGGACTACHVGGGTWTCTAAT |
| V4_3R | caagcagaagacggcatacgagat**GCCTAA**gtgactggagttcagacgtgtgctcttccgatctGGACTACHVGGGTWTCTAAT |
| V4_4R | caagcagaagacggcatacgagat**TGGTCA**gtgactggagttcagacgtgtgctcttccgatctGGACTACHVGGGTWTCTAAT |
| V4_5R | caagcagaagacggcatacgagat**CACTGT**gtgactggagttcagacgtgtgctcttccgatctGGACTACHVGGGTWTCTAAT |
| V4_6R | caagcagaagacggcatacgagat**ATTGGC**gtgactggagttcagacgtgtgctcttccgatctGGACTACHVGGGTWTCTAAT |
| V4_7R | caagcagaagacggcatacgagat**GATCTG**gtgactggagttcagacgtgtgctcttccgatctGGACTACHVGGGTWTCTAAT |
| V4_8R | caagcagaagacggcatacgagat**TCAAGT**gtgactggagttcagacgtgtgctcttccgatctGGACTACHVGGGTWTCTAAT |
| V4_9R | caagcagaagacggcatacgagat**CTGATC**gtgactggagttcagacgtgtgctcttccgatctGGACTACHVGGGTWTCTAAT |
| V4_10R | caagcagaagacggcatacgagat**AAGCTA**gtgactggagttcagacgtgtgctcttccgatctGGACTACHVGGGTWTCTAAT |

V4_11R caagcagaagacggcatacgagat**GTAGCC**gtgactggagttcagacgtgtgctcttccgatctGGACTACHVGGGTWTCTAAT

V4_12R caagcagaagacggcatacgagat**TACAAG**gtgactggagttcagacgtgtgctcttccgatctGGACTACHVGGGTWTCTAAT

V4_13R caagcagaagacggcatacgagat**CGTACT**gtgactggagttcagacgtgtgctcttccgatctGGACTACHVGGGTWTCTAAT

V4_14R caagcagaagacggcatacgagat**GACTGA**gtgactggagttcagacgtgtgctcttccgatctGGACTACHVGGGTWTCTAAT

V4_15R caagcagaagacggcatacgagat**GCTCAA**gtgactggagttcagacgtgtgctcttccgatctGGACTACHVGGGTWTCTAAT

V4_16R caagcagaagacggcatacgagat**TCGCTT**gtgactggagttcagacgtgtgctcttccgatctGGACTACHVGGGTWTCTAAT

**Supplementary Table S1.** Nucleotide sequences of primers used in the construction of libraries for Illumina sequencing. Lowercase letters denote adapter sequences necessary for binding to the flowcell, underlined lowercase are binding sites for the Illumina sequencing primers, bold uppercase highlight the index sequences (all the indexes were obtained from Illumina) and regular uppercase are the V3 and V4 region primers (341F on for the forward primer and 806R for the reverse primers). The inclusion of four maximally degenerated bases (“NNNN”) maximizes diversity during the first four bases of the run. Diversity is important for identifying unique clusters and base-calling accuracy [11, 12].

[11] Bokulich NA, Joseph CM, Allen G, Benson AK, Mills DA (2012) Next-generation sequencing reveals significant bacterial diversity of botrytized wine. PLoS One 7:e36357, DOI:10.1371/journal.pone.0036357

[12] Bartram AK, Lynch MD, Stearns JC, Moreno-Hagelsieb G, Neufeld JD (2011) Generation of multimillion-sequence 16S rRNA gene libraries from complex microbial communities by assembling paired-end illumina reads. Appl Environ Microbiol 77:3846-3852, DOI:10.1128/AEM.02772-10
