# Supplementary figures and images for: Intestinal microbiota and anastomotic leakage of stapled colorectal anastomoses: a pilot study
Source: Surg Endosc. 2015 Sep 18;30:2259–65. doi: 10.1007/s00464-015-4508-z (PMC4887536; doi:10.1007/s00464-015-4508-z)

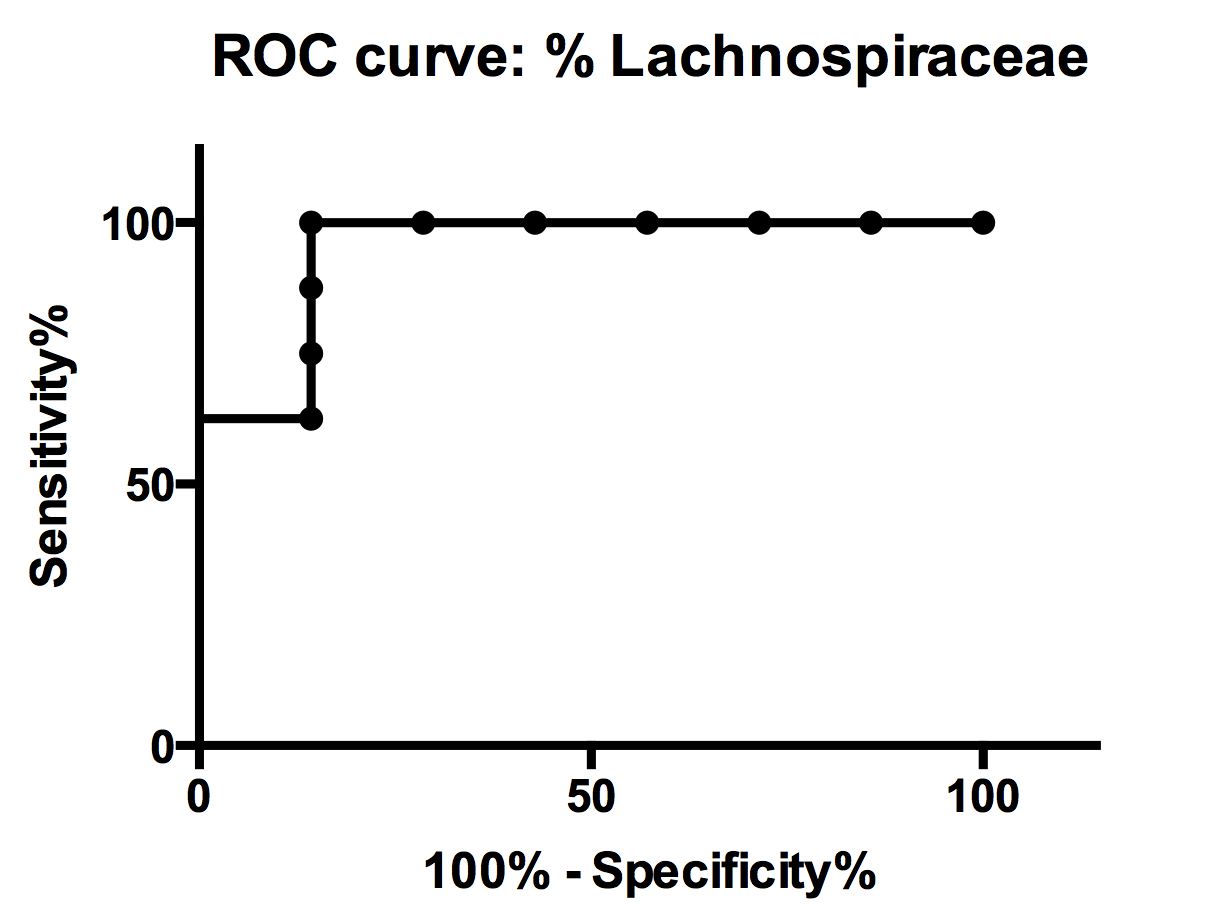

Supplement: Supplementary file 2 — Supplementary material 2 (TIFF 72 kb) [file 464_2015_4508_MOESM2_ESM.tiff]

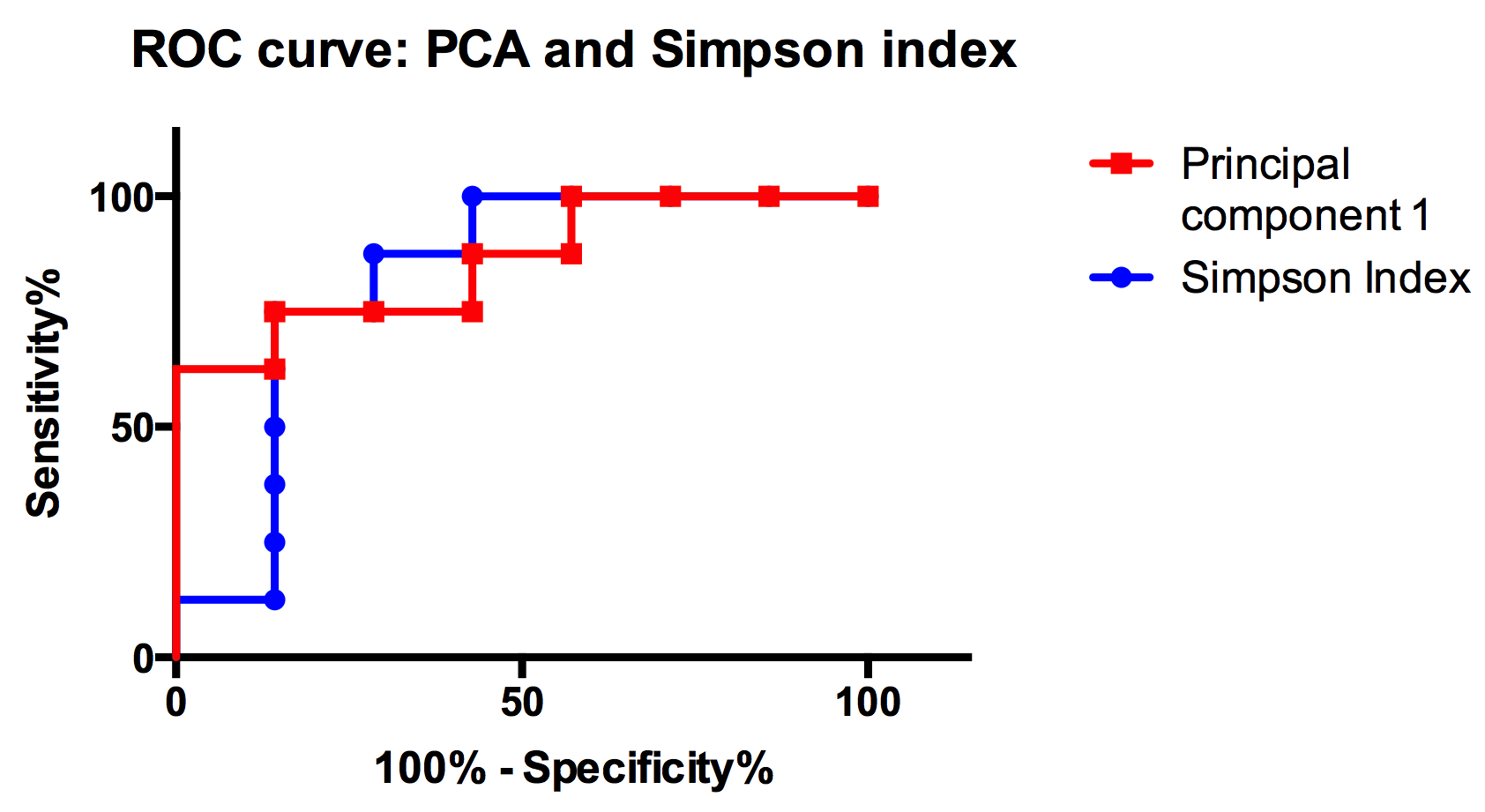

Supplement: Supplementary file 3 — Supplementary material 3 (TIFF 108 kb) [file 464_2015_4508_MOESM3_ESM.tiff]
